# Selecting the optimal risk threshold of diabetes risk scores to identify high-risk individuals for diabetes prevention: a cost-effectiveness analysis

**DOI:** 10.1007/s00592-019-01451-1

**Published:** 2019-11-19

**Authors:** Kristin Mühlenbruch, Xiaohui Zhuo, Barbara Bardenheier, Hui Shao, Michael Laxy, Andrea Icks, Ping Zhang, Edward W. Gregg, Matthias B. Schulze

**Affiliations:** 1 Department of Molecular Epidemiology, German Institute of Human Nutrition Potsdam-Rehbruecke, Nuthetal, Germany; 2 German Center for Diabetes Research (DZD), Neuherberg, Germany; 3 Division of Diabetes Translation, National Center for Chronic Disease Prevention and Health Promotion, Centers for Disease Control and Prevention, Atlanta, GA USA; 4 Helmholtz Zentrum München, German Research Center for Environmental Health (GmbH), Institute of Health Economics and Health Care Management, Neuherberg, Germany; 5 Institute of Health Services Research and Health Economics, German Diabetes Centre, Leibniz-Centre for Diabetes Research, Düsseldorf, Germany; 6 Institute of Health Services Research and Health Economics, Medical Faculty, Heinrich-Heine-University, Düsseldorf, Germany; 7 Institute of Nutritional Sciences, University of Potsdam, Potsdam, Germany

**Keywords:** Diabetes mellitus, Type 2, Cost-effectiveness analysis, Lifestyle risk reduction, Clinical prediction rule

## Abstract

**Aims:**

Although risk scores to predict type 2 diabetes exist, cost-effectiveness of risk thresholds to target prevention interventions are unknown. We applied cost-effectiveness analysis to identify optimal thresholds of predicted risk to target a low-cost community-based intervention in the USA.

**Methods:**

We used a validated Markov-based type 2 diabetes simulation model to evaluate the lifetime cost-effectiveness of alternative thresholds of diabetes risk. Population characteristics for the model were obtained from NHANES 2001–2004 and incidence rates and performance of two noninvasive diabetes risk scores (German diabetes risk score, GDRS, and ARIC 2009 score) were determined in the ARIC and Cardiovascular Health Study (CHS). Incremental cost-effectiveness ratios (ICERs) were calculated for increasing risk score thresholds. Two scenarios were assumed: 1-stage (risk score only) and 2-stage (risk score plus fasting plasma glucose (FPG) test (threshold 100 mg/dl) in the high-risk group).

**Results:**

In ARIC and CHS combined, the area under the receiver operating characteristic curve for the GDRS and the ARIC 2009 score were 0.691 (0.677–0.704) and 0.720 (0.707–0.732), respectively. The optimal threshold of predicted diabetes risk (ICER < $50,000/QALY gained in case of intervention in those above the threshold) was 7% for the GDRS and 9% for the ARIC 2009 score. In the 2-stage scenario, ICERs for all cutoffs ≥ 5% were below $50,000/QALY gained.

**Conclusions:**

Intervening in those with ≥ 7% diabetes risk based on the GDRS or ≥ 9% on the ARIC 2009 score would be cost-effective. A risk score threshold ≥ 5% together with elevated FPG would also allow targeting interventions cost-effectively.

**Electronic supplementary material:**

The online version of this article (10.1007/s00592-019-01451-1) contains supplementary material, which is available to authorized users.

## Introduction

Diabetes risk scores allow calculation of predicted risk based on several individual characteristics. However, using risk scores as a screening and risk stratification tool requires decisions about specific thresholds of predicted risk whereby individuals should be referred for intervention. Selecting such thresholds is difficult given that risk scores have a continuous association with diabetes risk. Cost-effectiveness analysis provides a framework for identifying the economically optimal threshold from the perspective of efficiently using health care resources. Using cost-effectiveness analysis to identify the economically optimal threshold for diabetes prevention has been applied to fasting glucose [1], HbA_1c_ [2] and a combination of glucose testing and risk scores [3, 4]; however, noninvasive risk scores for type 2 diabetes do not require blood sampling and can therefore be useful tools to guide providers whether a diagnostic blood test for prediabetes be performed [5]. We are not aware of studies applying cost-effectiveness analysis to the application of diabetes risk scores alone or to a two-step screening procedure as described above.

The aim of this study was to apply the framework of cost-effectiveness analysis to identify optimal thresholds of predicted risk from noninvasive diabetes risk scores to target a low-cost community-based intervention. We considered two screening scenarios: a 1-stage scenario with risk score assessment only and a 2-stage scenario in which the risk score assessment is followed by a fasting plasma glucose testing.

## Methods

### General methodological concept

**Fig. 1:**
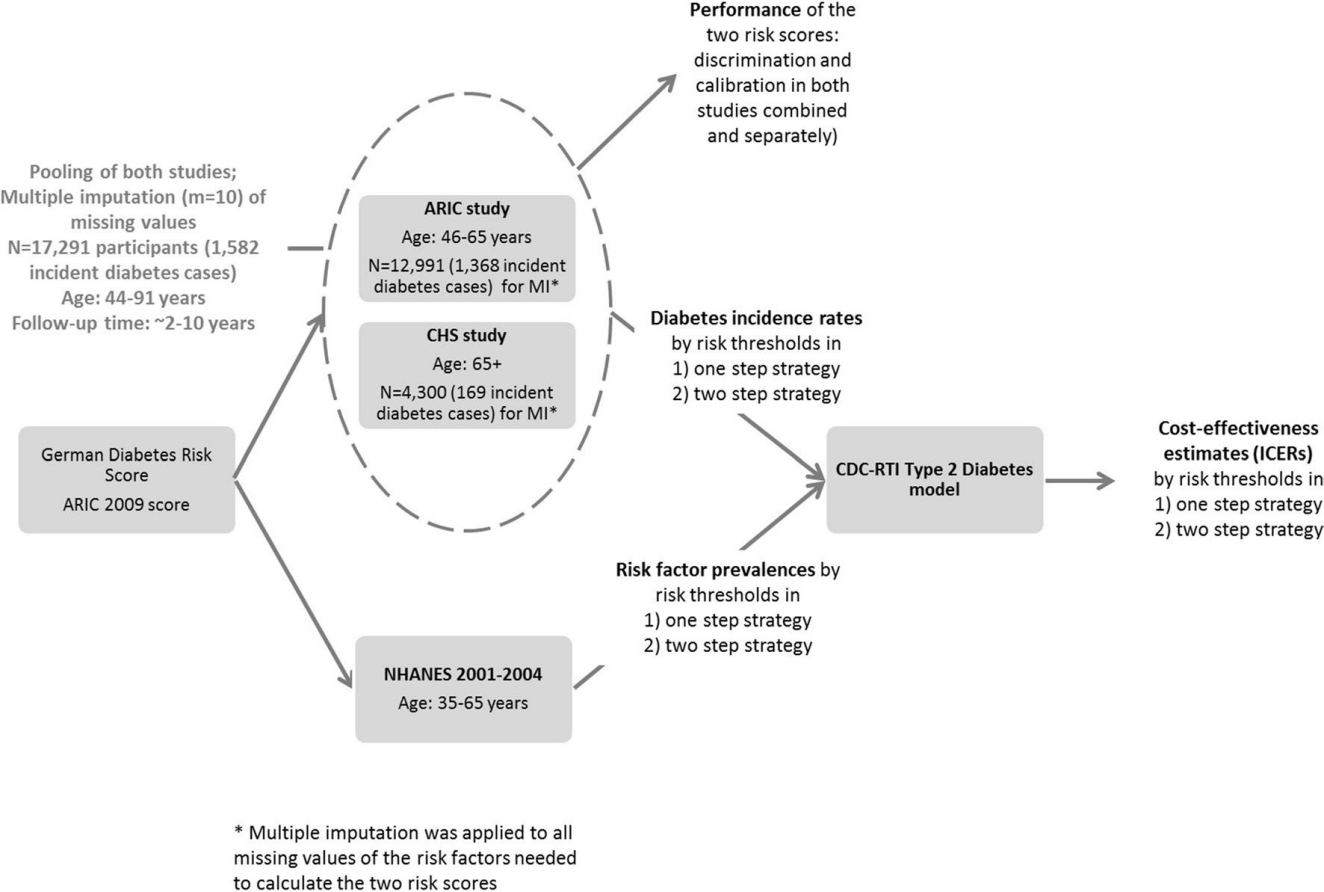
Conceptual overview of the elements and different steps of the methods applied in this study

Figure 1 illustrates the general methodological concept. We simulated the long-term cost-effectiveness of prevention intervention in high-risk individuals in the context of the US population. For defining the high-risk target group for intervention, we examined two scenarios: (1) a one-step screening strategy with a risk score only; and (2) a two-step screening strategy wherein a fasting glucose test (threshold 100 mg/dl—the cutoff for impaired fasting glucose [5]) followed among individuals that were screened positive with the risk score. This procedure included the following steps: (a) evaluation of diabetes risk scores in a combined dataset of the Atherosclerosis Risk in Communities (ARIC) study and Cardiovascular Health Study (CHS) to determine model performance and diabetes incidence rates by different risk thresholds, (b) evaluation of risk factor prevalences by risk thresholds using National Health and Nutrition Examination Survey (NHANES) 2001–2004 and (c) applying estimates from (a) and (b) in the Centers for Disease Control and Prevention/Research Triangle Institute (CDC/RTI) Diabetes Cost-effectiveness Model to calculate the incremental cost-effectiveness ratios (ICER) as additional cost per quality adjusted life-year (QALY) gained across alternative thresholds of predicted 5-year diabetes risk calculated using the diabetes risk scores under the two scenarios. A risk threshold would be selected as economically optimal if associated with the highest QALY gain while being < $50,000/QALY gained [6–8].

### Diabetes risk scores

We considered diabetes risk scores which were (a) based on noninvasively assessable risk factors, (b) for which performance has been validated in varying populations and (c) for which scoring algorithms were available. Based on a systematic review [9], we selected the ARIC 2009 score [10] which was developed in a US population. Although an additional US based diabetes risk score (Framingham Offspring Study) met our criteria, this score had limited accuracy in validation studies [9, 11], specifically in other US cohorts [12]. As second risk score, we considered the German Diabetes Risk Score (GDRS) [13–15] which showed comparable accuracy in validation studies to the ARIC 2009 score [11]. By comparing these scores, we are able to evaluate whether results are risk score specific or more general.

To determine the performance of the two diabetes risk scores in a US population, individual 5-year predicted diabetes risks were calculated in the ARIC and CHS studies based on published equations [10, 13]. Due to a large amount of missing information for the risk score components, multiple imputation was performed. Based on 10 imputed datasets, the average discrimination was evaluated by the area under the receiver operating characteristic curve (ROC-AUC) [16].

### Diabetes incidence according to thresholds of predicted diabetes risk

The diabetes incidence rates for the US population were derived from the ARIC and CHS studies. A detailed description of the study populations and how data were combined is reported in the Online Resource 1. Based on 1582 incident diabetes cases within a follow-up time of ~ 2–10 years from 10 multiple imputation datasets, diabetes incidence rates were determined for a series of high-risk cohorts defined with a range of thresholds of predicted 5-year diabetes risk with GDRS and ARIC 2009 score. For the 2-stage screening scenario, incidence rates were determined among those subgroups identified to be high-risk from the risk scores which had additionally fasting glucose ≥ 100 mg/dl.

### Overview of the CDC-RTI type 2 diabetes model

The CDC Type 2 Diabetes Cost-effectiveness Model was designed to simulate the development and progression of type 2 diabetes to assess the cost-effectiveness of various prevention and treatment interventions. The basic model has been described and validated elsewhere [17–20]. Briefly, it is a Markov simulation model of disease progression and cost-effectiveness that follows persons from the onset of disease until death or age 95. In the model, separate modules are used to simulate the development of type 2 diabetes, hypertension, hyperlipidemia, coronary heart diseases, and stroke among high-risk individuals. For individuals who developed type 2 diabetes, the model additionally simulates three diabetes-related microvascular complications (nephropathy, neuropathy, and retinopathy), which are primarily based upon observations of the UK Prospective Diabetes Study [21]. Model outcomes include disease complications, death, costs, and QALYs. The model has been validated and used for assessing the lifetime cost-effectiveness of various interventions for preventing type 2 diabetes and its complications (19–24).

### Perspective, cost and utilities

The study was conducted from a health care system perspective and included the costs of the lifestyle intervention, health clinic visits, risk assessment and FPG testing (Table 1), and direct medical costs. We assumed a one-off screening scenario with 100% coverage in the population. Risk assessment is attached to a visit in a health clinic and performed by the patient. For the two-stage screening scenario, the blood test follows only for patients with an increased risk as defined by the risk thresholds analyzed. Screen-detected diabetes cases were not considered in the simulation. Among individuals who did not develop diabetes, direct medical costs included medical costs of treating hypertension, hyperlipidemia, stroke, and coronary heart diseases. For individuals who developed type 2 diabetes, the direct medical costs also included diabetes treatment cost and the costs of treating diabetes-related complications [22, 23]. The annual health utility was estimated for each cohort each year using the equation developed by Coffey et al. [24]. The implementation of diabetes interventions and occurrence of complications had different effects on patients’ health utility. For example, the use of insulin was associated with − 0.034 health utility decrement, and the development of blindness would reduce the health utility score by 0.043.

**Table 1:** Assumptions for the simulation model regarding cost and effectiveness parameters

	Base-case 1-stage and 2-stage	Deterministic sensitivity analyses 1-stage and 2-stage
Cost of screening instrument		Not varied
Risk score	$0 (assumed)	
Glucose test	$5.01 (Medicare fee schedule 2011)	
Cost of additional time in physician office visits		Not varied
Risk score	$0 (assumed)	
Glucose test	$53.2 (Medicare fee schedule 2011)	
Percentage participating in lifestyle intervention	50% (assumed)	Not varied
Percentage completing lifestyle intervention	50% Burke et al. [26] and Gans et al. [27]	Not varied
Intervention	Group-based lifestyle intervention at community level (Y-DPP)	
Diabetes risk reduction in first 3 years	25%	12.5% (SA2)/50% (SA3)/stable over time
After 3 years	12.5% (assumed as half of original DPP)	6.75% (SA2)/25% (SA3)
Hypertension risk reduction	0% (assumed)	(SA5)
Hypercholesterolemia risk reduction	0% (assumed)	Not varied
Lifestyle Intervention cost	–	Stable over time (SA4)
Year 1	$375 Ackermann et al. [25]	750$ (SA1)
Year 2	$375 (assumed)	750$ (SA1)
3 and after	$375 (assumed)	750$ (SA1)
Impact of intervention on medical costs	$0 (assumed)	Not varied
Utility		Not varied
Score 0.05–0.20	Coffey’s model [24]	Not varied
Diabetes	Coffey’s model [24]	Not varied
CVD before diabetes	NHANES (2001–2004)	Not varied
Microvascular complications before diabetes	NHANES (2001–2004)	Not varied
Hypertension before diabetes	NHANES (2001–2004)	Not varied
Hypercholesterolemia before diabetes	NHANES (2001–2004)	Not varied

### Parameters of the model

The simulation sample was derived from data on non-diabetic US adults aged 35–65 years in NHANES 2001–2004. According to both the 1-stage and 2-stage approach for the two risk scores, we created multiple cohorts stratified by individuals’ demographics and risk factors including age, sex, race/ethnicity, hypertension, smoking, and total cholesterol and accounted for the joint distribution of those variables.

We applied a low-cost community-based intervention similar to the Y-DPP (Diabetes Prevention Program) [25]. We assumed the program leads to a 25% risk reduction in 3 years which diminishes to 12.5% after year 3 and is maintained thereafter. These estimates include a participation rate of 50% and a compliance rate of 50% was assumed in the intervention group [26, 27].

ICERs were calculated by dividing incremental costs measured in 2012 US dollars by incremental health benefit measured by QALYs. ICERs were expressed in 2012 US dollars. Both future health benefits and costs were discounted at an annual rate of 3% [28]. To identify the economically optimal threshold of predicted diabetes risk, we calculated ICERs for different thresholds of predicted diabetes risk for the two risk scores for both the 1-stage approach and the 2-stage approach.

We performed several one-way deterministic sensitivity analyses to examine how the cost-effectiveness results would change under different cost and effectiveness scenarios of the lifestyle intervention. To do so, we rerun analyses by changing the value of one parameter at a time while keeping all other parameters at their base-case values (Table 1). First, we doubled the cost of the lifestyle intervention to test whether a costlier intervention program might change the ICERs and thus the selection of the economically optimal cutoff points under each screening scenario. Second, we halved the diabetes risk reduction in the intervention to 12.5% in the first 3 years and to 6.75% in the years thereafter. Third, we doubled the risk reductions in the intervention from their base-case values. Fourth, we assumed costs and effectiveness of the lifestyle intervention to be stable over time. Finally, we added the potential additional benefit of the intervention on hypertension risk reduction.

We also performed probabilistic sensitivity analysis to generate the cost-effectiveness acceptability curve as recommended by good research practices for cost-effectiveness analysis [29]. We selected 18 most critical parameters (e.g., effect and cost of diabetes prevention program) and varied them simultaneously in 500 iterations (Suppl. Table 1 in Online Resource 1). The incremental costs of each risk threshold were plotted against their incremental effects (QALY) to form cost-effectiveness plane with a diagonal willingness-to-pay line set at $50,000/QALY. In addition, the probabilities of being cost-effective given a range of willingness-to-pay levels were plotted to form a cost-effectiveness acceptability curve.

## Results

### Performance of risk scores

The ROC-AUC (95%-CI) of the GDRS and the ARIC 2009 score for prediction of incident diabetes in ARIC and CHS was 0.691 (0.677–0.704) and 0.720 (0.707–0.732), respectively. Comparable results were observed in ARIC, but lower ROC-AUC values were observed in CHS (Suppl. Table 2 in Online Resource 1). Sensitivity and specificity for varying risk thresholds for both scores are tabulated in Suppl. Table 3 in Online Resource 1. Including fasting glucose in addition, the risk scores in ARIC and CHS increased the ROC-AUC to 0.787 (0.774–0.799) for the GDRS and 0.800 (0.788–0.812) for the ARIC 2009 score.

### Base case analysis

Suppl. Table 4 in Online Resource 1 shows the annual incidence rates by threshold of predicted diabetes risk for both risk scores and for the two screening approaches. When comparing the rates directly for each risk threshold, we observed higher incidence rates for subgroups identified with the GDRS for thresholds up to 13% risk for the 1-stage and up to 12% for the 2-stage approach; for thresholds higher than these values, higher incidence rates were observed when the ARIC 2009 score was used to predict risk.

**Fig. 2:**
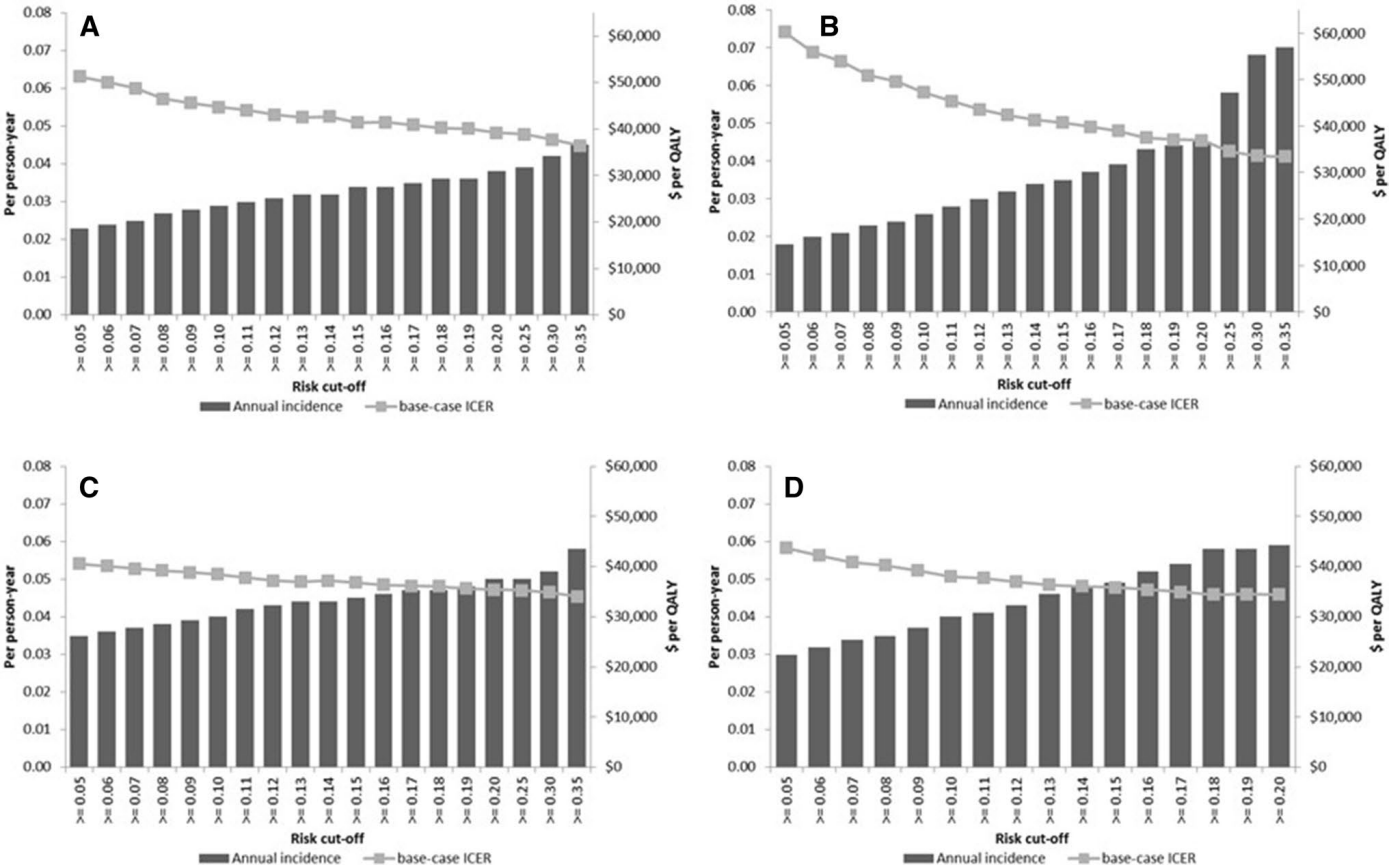
Annual incidence rates and cost per QALY gained (ICER) by risk cutoffs according to the GDRS (**a**, **c**) and the ARIC 2009 score (**b**, **d**) in a 1-stage (risk score only, **a**, **b**) and 2-stage screening approach (risk score and subsequent fasting plasma glucose **c**, **d**)

Figure 2 shows the cost per QALY associated with alternative thresholds of predicted diabetes risk. Overall, increasing the threshold of predicted diabetes risk was related to an increase in annual incidence rate and a decrease in cost per QALY gained. In the one-stage screening approach, a threshold of 5% predicted risk resulted in ICERs of $51,318/QALY gained for the GDRS and $60,170/QALY gained for the ARIC 2009 score. Increasing the threshold to 7% risk for the GDRS resulted in an ICER ($48,752/QALY gained) lower than the pre-specified cutoff of < $50,000/QALY gained. The threshold of the ARIC 2009 score for which ICER was < $50,000/QALY gained was 9% predicted diabetes risk. For the two-stage screening approach, ICER was < $50,000/QALY gained for all investigated thresholds of predicted diabetes risk (starting at 5%) for both risk scores. Results for all scenarios are additionally summarized in a cost-effectiveness plane (Suppl. Fig. 1 in Online Resource 1).

When evaluating NAHES 2001–2004 data, the thresholds of ≥ 7% (GDRS) and ≥ 9% (ARIC 2009) risk correspond to about 29% and 40% of the US adult population to be screened positively and thus being a target for intervention (Suppl. Table 5 in Online Resource 1). In the 2-stage screening scenario, a diabetes risk threshold of ≥ 5% risk in combination with impaired fasting glucose resulted in 20% of US adults identified for intervention when using the GDRS and 27% when using the ARIC 2009 score.

### Sensitivity analyses

Results from deterministic sensitivity analyses are displayed in Suppl. Fig. 2 in Online Resource 1. The strongest influence on the results was observed for parameters associated with the costs and effectiveness of the preventive intervention. Results from probabilistic sensitivity analyses are presented in Suppl. Figs. 3 and 4 in Online Resource 1. The probability for the proposed strategy being cost-effective varied by different willingness-to-pay thresholds. Based on a willingness-to-pay threshold of $50,000/QALY, $60,000/QALY, and $70,000/QALY, the probabilities for the proposed strategy being cost-effective were estimated to be 48.3%, 87.1%, and 98.9%.

## Discussion

Our results indicate that, from a health care system perspective, a low-cost community-based intervention is cost-effective in the USA when target groups are identified by noninvasive diabetes risk scores. Screening with the GDRS or the ARIC 2009 score allows cost-effective intervention at thresholds of 7% and 9% predicted 5-year diabetes risk, respectively. If additional glucose tests are feasible (2-step screening), lower thresholds of predicted diabetes risk can be applied to identify a high-risk group for cost-effective intervention. We have investigated two diabetes risk scores, and while the overall performance to predict incident diabetes was comparable, the observed differences in absolute risk thresholds identified for both scores suggest that individual scores need to be evaluated in detail before their application.

We used the commonly used cutoff of $50,000/QALY gained to define cost-effectiveness [6–8]. However, this cutoff is not universally considered optimal and others have been discussed [30]. Stakeholders and policy makers might be more comfortable with lower cost-effectiveness thresholds, as this usually results in smaller target groups and thus lower total costs of interventions. For example, using $40,000/QALY gained, the selected risk thresholds to be optimal would be considerably higher (16% and 20% risk for the ARIC 2009 score and the GDRS, respectively), resulting in a considerably smaller proportion of individuals qualifying for intervention.

Two previous studies evaluated cost-effectiveness of diabetes prevention in the context of screening with diabetes risk scores. Chen et al. [3] showed that costs were lowest for a 2-stage approach which involved the original AusDrisk risk score and a recalculation of risk with an extended risk score which additionally included fasting glucose. While, several risk thresholds were evaluated, the economically optimal threshold or the cost-effectiveness in terms of cost per QALY gained were not systematically investigated. Sullivan et al. [4] reported that a 2-stage strategy which additionally considered a diabetes risk score was more cost-effective than a screening strategy for identifying high-risk individuals based on impaired fasting glucose alone. However, the assumed intervention was not a lifestyle intervention and the risk score was not noninvasive but rather based on multiple biomarkers. Neither of the two studies investigated a comparable 1-stage screening scenario with a noninvasive risk score. Our analyses also extend previous publications which evaluated varying cutoffs of fasting glucose or HbA_1c_ as screening tool [1, 2]. Interestingly, the recommended cutoff for impaired fasting glucose (100 mg/dl) [5] was not cost-effective in the context of targeting the DPP intervention [1]. However, our results strongly support that initial risk score based screening to select individuals for further fasting glucose testing considerably increases cost-effectiveness. Using conventional risk factors and fasting glucose together for prediction has been shown to outperform prediction based on conventional risk factors or fasting glucose only both for ARIC and the GDRS [31, 32], further supported by our results. Thus, if applicable, a two-stage screening approach is preferable.

Our study has several limitations. First, our analyses were based on various assumptions for the simulation model. The low-cost lifestyle intervention program considered represents a group-based intervention in the communities based on DPP. Group-based interventions were shown to achieve the same effectiveness as individual programs or to be cost-effective before [18, 33, 34]. Also, the 4.4% weight loss observed in translational programs from the US National DPP in the first year [35] can be translated to a 35.4% risk reduction. Based on this evidence, the assumed intervention effect (25% risk reduction) seems reasonable. Still, we cannot rule out that effectiveness might be heterogeneous in high-risk groups according to different patient characteristic such as age, sex, family history of diabetes, or other risk factors. However, given that lifestyle intervention among individuals with prediabetes appear to be more effective among those with higher diabetes risk based on a noninvasive risk score [36], we believe our assumed intervention effect is rather conservative. We furthermore addressed uncertainty about this assumption in several sensitivity analyses. Although the diabetes incidence rates according to thresholds of diabetes risk are based on the large, fairly representative cohort studies ARIC and CHS, incidence could vary in different populations. Furthermore, we did not evaluate thresholds of diabetes risk below 5% for both risk scores. Our results indicate here that cutoffs lower than 5% might still be cost-effective if screening by fasting glucose follows initial risk score screening. In addition, the cost model has specifically been developed for cost-effectiveness analyses in a US context; generalizability of our findings to other countries is therefore unclear. We also assumed a one-off screening strategy for our simulation, but repetitive screening might change overall cost-effectiveness and thereby the optimal risk thresholds. Moreover, we assumed a screening scenario with 100% coverage in the population, and future studies are needed to evaluate different screening scenarios.

In conclusion, our findings suggest that noninvasive diabetes risk scores, such as the GDRS or the ARIC 2009 score, allow identification of high-risk target groups for cost-effective lifestyle interventions to prevent type 2 diabetes. The findings specifically support economically optimal thresholds of predicted risk derived from these risk scores for targeting community-based lifestyle interventions under a US healthcare system perspective. Such thresholds can be used to justify categories of risk when risk scores are used as tests in clinical practice. Our finding, that risk score based screening followed by fasting glucose testing increases cost-effectiveness supports current recommendations to use risk test to guide providers on whether performing a diagnostic test for prediabetes [5].

## Electronic supplementary material

Below is the link to the electronic supplementary material.

Supplementary material 1 (DOCX 1001 kb)
