# Supplementary material for: Selecting the optimal risk threshold of diabetes risk scores to identify high-risk individuals for diabetes prevention: a cost-effectiveness analysis
Source: Acta Diabetol. 2019 Nov 19;57(4):447–54. doi: 10.1007/s00592-019-01451-1 (PMC7093341; doi:10.1007/s00592-019-01451-1)
Supplement: Supplementary file 1 — Supplementary material 1 (DOCX 1001 kb) [file 592_2019_1451_MOESM1_ESM.docx]

**Electronic Supplementary Material**

**Selecting the optimal risk threshold of diabetes risk scores to identify high-risk individuals for diabetes prevention: a cost-effectiveness analysis**

Acta Diabetologia

Kristin Mühlenbruch , Xiaohui Zhuo, Barbara Bardenheier, Hui Shao, Michael Laxy, Andrea Icks, Ping Zhang, Edward W. Gregg, Matthias B. Schulze*

* German Institute of Human Nutrition Potsdam-Rehbruecke, Email: mschulze@dife.de

**Suppl. Appendix. Description of the Study Population**

**Suppl. Table 1. Input parameters for probabilistic sensitivity analysis**

**Suppl. Table 2. Discrimination of the GDRS and the ARIC 2009 score in the ARIC study, CHS study and combined.**

**Suppl. Table 3. Sensitivity and specificity of varying thresholds of absolute risk estimated by the GDRS and the ARIC 2009 score in the ARIC and CHS studies**

**Suppl. Table 4. Population size according to thresholds of the 5-year diabetes risk determined with the GDRS and ARIC 2009 score for a 1-stage and a 2-stage screening scenario.**

**Suppl. Table. 5 Population size according to thresholds of the 5-year diabetes risk determined with the GDRS and ARIC 2009 score for a 1-stage and a 2-stage screening scenario.**

**Suppl. Fig. 1 Cost-effectiveness Plane for all scenarios of the base-case analysis.**

**Suppl. Fig. 2 Annual incidence rates and cost per QALY gained (ICER) by risk cut-offs of the GDRS and the ARIC 2009 model in a 1-stage and 2-stage appraoch for five different sensitivity analyses.**

**Suppl. Fig. 3 Scatter Plot for Probabilistic Sensitivity Analysis**

**Suppl. Fig. 4 Probabilistic Acceptance Curve**

**Suppl. Appendix. Description of the Study Population**

The details of the study designs of ARIC and CHS have been described elsewhere ([1](#_ENREF_1); [2](#_ENREF_2)). Briefly, the ARIC study is a large-scale long-term prospective study including 15,792 black and white men and women aged 46 to 65 years from four diverse communities. The baseline assessment (cycle 1) included a home interview, a clinical examination and blood drawing after 12 hours of fasting. Based on this, socio-demographic, lifestyle and anthropometric risk factors, family history of diseases, medication use and biochemical parameters were obtained. Follow-up questionnaires were sent out annually and detailed examinations were organized in 3-year cycles (2, 3, 4). For the current study, data from cycles 1, 2 and 3 were used with incident diabetes definition from cycles 2 and 3. After exclusion of prevalent diabetes cases or missing prevalent diabetes status or high fasting glucose 12,991 participants were available in a multiple imputation procedure for missing risk factor information needed in the risk scores. Within a follow-up time of 2 to 9 years, an average of 1,368 incident diabetes cases was observed across all 10 imputation sets.

The CHS study is a population-based prospective cohort study in the elderly designed to study risk factors for the onset and progression of coronary heart disease and stroke. The study included 5,888 participants who were recruited from four different communities in the US; 5,201 participants were included in the initial cohort and 687 participants in the new cohort, predominantly being of African-American ethnicity. At baseline examination, a home interview and clinic examination was conducted to assess psychosocial factors, medication, anthropometry and lifestyle factors as well as blood parameters and extensive clinic examination for identifying subclinical disease states. The 2,962 women and 2,239 men aged 65 years and older were followed up annually by telephone or clinic visit if necessary and extensive re-examination was conducted 3 years after baseline assessment. For the current analysis, follow-up from year 5 was used to determine incident diabetes status. After exclusion of prevalent or missing diabetes status or high fasting glucose, 4,300 participants were used in the multiple imputation process. Between 2 to 8 years of follow-up, on average169 incident diabetes cases occurred across 10 imputed sets of data.

To determine the performance of the two diabetes risk scores and the diabetes incidence rates for varying cut-offs of the scores - valid for a general adult US population - we combined the ARIC and CHS studies and ended up with 17,291 participants for multiple imputation of missing risk factor information ([3-5](#_ENREF_3)) within an age range of 44 to 91 years. The fraction of missingness ranged from 0% to 25% for the risk factors or the outcome of the two risk scores; after application of MI using Markov Chain Monte Carlo method, a relative efficiency of 94% to 99.9% could be achieved with 10 imputations confirming the validity of this approach.

The NHANES are a series of nationwide consecutive representative cross-sectional surveys of the non-institutionalized US population. NHANES, conducted by NCHS of the Centers for Disease Control and Prevention (CDC), have a stratified multistage probability design. Minority groups of certain ethnicities, such as non-Hispanic black Americans and Mexican Americans, certain ages, and certain income levels were oversampled to make stable estimates for these groups. NHANES comprised in-home interviews and health examinations in a mobile examination center. NHANES 2001-2002 included 11039 participants and NHANES 2003-2004 included 10122 participants (6).

1. The Atherosclerosis Risk in Communities (ARIC) Study: design and objectives. The ARIC investigators. American journal of epidemiology 1989;129:687-702

2. Fried LP, Borhani NO, Enright P, Furberg CD, Gardin JM, Kronmal RA, Kuller LH, Manolio TA, Mittelmark MB, Newman A, et al.: The Cardiovascular Health Study: design and rationale. Annals of epidemiology 1991;1:263-276

3. Marshall A, Altman DG, Holder RL, Royston P: Combining estimates of interest in prognostic modelling studies after multiple imputation: current practice and guidelines. BMC medical research methodology 2009;9:57

4. Rubin DB: Multiple Imputation for Nonresponse in Surveys. New York, J. Wiley & Sons, 1987

5. Sterne JA, White IR, Carlin JB, Spratt M, Royston P, Kenward MG, Wood AM, Carpenter JR: Multiple imputation for missing data in epidemiological and clinical research: potential and pitfalls. BMJ 2009;338:b2393

6. https://wwwn.cdc.gov/nchs/nhanes/ResponseRates.aspx#population-totals

| **Suppl. Table 1. Input parameters for probabilistic sensitivity analysis** | | | | | | |
| --- | --- | --- | --- | --- | --- | --- |
| **Variables** |  | **Distributions** |  | **Control Group** | | |
|  |  |  |  | Lower Bound |  | Upper Bound |
| *Control Group* |  |  |  |  |  |  |
| Minimum HbA_1c_ Level (%) |  | Uniform |  | 5.80 |  | 6.20 |
| Maximum HbA_1c_ Level (%) |  | Uniform |  | 10.00 |  | 12.00 |
| Initial HbA_1c_ Level (%) |  | Uniform |  | 6.30 |  | 6.70 |
| Rate of HbA_1c_ Change per year (%) |  | Uniform |  | 0.05 |  | 0.07 |
| Treatment Effect on HbA_1c_ (%) |  | Uniform |  | 0.00 |  | 0.00 |
| Risk Reduction (%) year 1~3 |  | Uniform |  | 0.00 |  | 0.00 |
| Risk Reduction (%) year 4+ |  | Uniform |  | 0.00 |  | 0.00 |
| DPP Cost ($) year 1 |  | Uniform |  | 41.25 |  | 68.75 |
| DPP Cost ($) year 2+ |  | Uniform |  | 17.25 |  | 28.75 |
| *Treatment Group* |  |  |  |  |  |  |
| Minimum HbA_1c_ Level (%) |  | Uniform |  | 5.80 |  | 6.20 |
| Maximum HbA_1c_ Level (%) |  | Uniform |  | 8.00 |  | 10.00 |
| Initial HbA_1c_ Level (%) |  | Uniform |  | 6.30 |  | 6.70 |
| Rate of HbA_1c_ Change per year (%) |  | Uniform |  | 0.05 |  | 0.07 |
| Treatment Effect on HbA_1c_ (%) |  | Uniform |  | 0.70 |  | 1.10 |
| Risk Reduction (%) year 1~3 |  | Uniform |  | 18.75 |  | 31.25 |
| Risk Reduction (%) year 4+ |  | Uniform |  | 7.50 |  | 12.50 |
| DPP Cost ($) year 1~3 |  | Uniform |  | 300.00 |  | 500.00 |
| DPP Cost ($) year 4+ |  | Uniform |  | 262.50 |  | 437.50 |

**Suppl. Table 2. Discrimination of the GDRS and the ARIC 2009 score in the ARIC study, CHS study and combined.**

|  | **ROC-AUC (95%-CI)** | |
| --- | --- | --- |
|  | **GDRS** | **ARIC 2009** |
| ARIC study | 0.719 (0.705-0.733) | 0.730 (0.717-0.744) |
| CHS study | 0.629 (0.588-0.670) | 0.630 (0.588-0.671) |
| Combined | 0.691 (0.677-0.704) | 0.720 (0.707-0.732) |

ARIC, Atherosclerosis Risk in Communities; CHS, Cardiovascular Health Study; GDRS, German Diabetes Risk Score; ROC-AUC, Area under the receiver-operating characteristic curve

**Suppl. Table 3. Sensitivity and specificity of varying thresholds of absolute risk estimated by the GDRS and the ARIC 2009 score in the ARIC and CHS studies**

| **Threshold of diabetes risk (≥)** | **GDRS** | | **ARIC 2009 score** | |
| --- | --- | --- | --- | --- |
|  | Sensitivity (%) | Specificity (%) | Sensitivity (%) | Specificity (%) |
| 0.05 | 67.6 | 53.4 | 95.4 | 19.4 |
| 0.06 | 62.8 | 59.6 | 92.2 | 29.0 |
| 0.07 | 59.0 | 64.4 | 88.0 | 36.8 |
| 0.08 | 54.8 | 68.3 | 83.7 | 44.8 |
| 0.09 | 51.3 | 71.9 | 78.2 | 52.5 |
| 0.10 | 48.3 | 74.8 | 73.0 | 59.3 |
| 0.11 | 45.4 | 77.2 | 68.4 | 64.2 |
| 0.12 | 43.1 | 79.3 | 64.1 | 68.8 |
| 0.13 | 40.5 | 81.2 | 57.4 | 73.8 |
| 0.14 | 38.4 | 82.7 | 53.6 | 77.3 |
| 0.15 | 36.8 | 84.2 | 48.1 | 80.5 |
| 0.16 | 34.8 | 85.3 | 43.4 | 83.7 |
| 0.17 | 32.6 | 86.4 | 39.4 | 85.9 |
| 0.18 | 30.8 | 87.5 | 34.8 | 88.7 |
| 0.19 | 29.5 | 88.5 | 30.5 | 90.4 |
| 0.20 | 27.7 | 89.3 | 26.8 | 91.7 |
| 0.25 | 21.6 | 92.3 | 13.2 | 97.0 |
| 0.30 | 17.4 | 94.2 | 6.8 | 98.7 |
| 0.35 | 14.1 | 95.6 | 2.9 | 99.5 |

ARIC, Atherosclerosis Risk in Communities; CHS, Cardiovascular Health Study; GDRS, German Diabetes Risk Score

**Suppl. Table 4. Annual Diabetes Incidence Rates from ARIC and CHS study data by GDRS and ARIC 2009 thresholds of the 5-year diabetes risk, the 1-stage and 2-stage approach for selecting the intervention group.**

| **Threshold of diabetes risk (≥)** | **Annual diabetes incidence rates** | | | |
| --- | --- | --- | --- | --- |
|  | **1-stage approach** | | **2-stage approach** | |
|  | GDRS | ARIC 2009 | GDRS | ARIC 2009 |
| 0.05 | 0.023 | 0.018 | 0.035 | 0.030 |
| 0.06 | 0.024 | 0.02 | 0.036 | 0.032 |
| 0.07 | 0.025 | 0.021 | 0.037 | 0.034 |
| 0.08 | 0.027 | 0.023 | 0.038 | 0.035 |
| 0.09 | 0.028 | 0.024 | 0.039 | 0.037 |
| 0.10 | 0.029 | 0.026 | 0.040 | 0.040 |
| 0.11 | 0.03 | 0.028 | 0.042 | 0.041 |
| 0.12 | 0.031 | 0.03 | 0.043 | 0.043 |
| 0.13 | 0.032 | 0.032 | 0.044 | 0.046 |
| 0.14 | 0.032 | 0.034 | 0.044 | 0.048 |
| 0.15 | 0.034 | 0.035 | 0.045 | 0.049 |
| 0.16 | 0.034 | 0.037 | 0.046 | 0.052 |
| 0.17 | 0.035 | 0.039 | 0.047 | 0.054 |
| 0.18 | 0.036 | 0.043 | 0.047 | 0.058 |
| 0.19 | 0.036 | 0.044 | 0.048 | 0.058 |
| 0.20 | 0.038 | 0.045 | 0.050 | 0.059 |
| 0.25 | 0.039 | 0.058 | 0.050 | 0.079 |
| 0.30 | 0.042 | 0.068 | 0.052 | 0.093 |
| 0.35 | 0.045 | 0.07 | 0.058 | 0.095 |

ARIC, Atherosclerosis Risk in Communities; CHS, Cardiovascular Health Study; GDRS, German Diabetes Risk Score

**Suppl. Table. 5 Population size according to thresholds of the 5-year diabetes risk determined with the GDRS and ARIC 2009 score for a 1-stage and a 2-stage screening scenario, NHANES 2001-2004.**

| **Threshold of the 5-year diabetes risk** | **Population Size N (%^a^)** | | | | |
| --- | --- | --- | --- | --- | --- |
|  | **1-stage approach** | |  | **2-stage approach** | |
|  | GDRS | ARIC 2009 score |  | GDRS | ARIC 2009 score |
| >= 0.05 | 1630 (37.07) | 2613 (62.33) |  | 179 (19.95) | 227 (26.88) |
| >= 0.06 | 1447 (32.70) | 2344 (55.19) |  | 169 (18.81) | 218 (25.50) |
| >= 0.07 | 1299 (29.35) | 2128 (49.82) |  | 152 (16.57) | 208 (24.31) |
| >= 0.08 | 1192 (26.68) | 1919 (44.96) |  | 142 (15.37) | 192 (22.67) |
| >= 0.09 | 1090 (24.34) | 1727 (39.97) |  | 122 (13.55) | 178 (20.87) |
| >= 0.10 | 1005 (22.20) | 1566 (36.05) |  | 111 (12.20) | 164 (18.76) |
| >= 0.11 | 930 (20.39) | 1423 (32.27) |  | 106 (11.64) | 157 (17.76) |
| >= 0.12 | 873 (19.08) | 1252 (27.65) |  | 103 (11.49) | 137 (14.87) |
| >= 0.13 | 819 (17.65) | 1093 (23.69) |  | 94 (10.42) | 120 (13.40) |
| >= 0.14 | 764 (16.49) | 973 (20.59) |  | 90 (10.15) | 108 (11.98) |
| >= 0.15 | 712 (15.42) | 880 (18.62) |  | 81 (9.01) | 98 (11.11) |
| >= 0.16 | 676 (14.60) | 764 (16.04) |  | 77 (8.59) | 87 (9.37) |
| >= 0.17 | 640 (13.72) | 663 (13.77) |  | 75 (8.45) | 76 (7.99) |
| >= 0.18 | 609 (13.08) | 563 (11.54) |  | 74 (8.35) | 70 (7.40) |
| >= 0.19 | 585 (12.52) | 498 (10.06) |  | 71 (8.21) | 63 (6.68) |
| >= 0.20 | 560 (11.85) | 431 (8.66) |  | 68 (7.62) | 51 (5.39) |
| >= 0.25 | 433 (8.91) | 189 (3.45) |  | 54 (6.06) | - |
| >= 0.30 | 347 (7.12) | 94 (1.7) |  | 47 (5.11) | - |
| >= 0.35 | 305 (6.37) | 32 (0.54) |  | 41 (4.39) | - |

ARIC, Atherosclerosis Risk in Communities; CHS, Cardiovascular Health Study; GDRS, German Diabetes Risk Score; NHANES, National Health and Nutrition Examination Survey

**
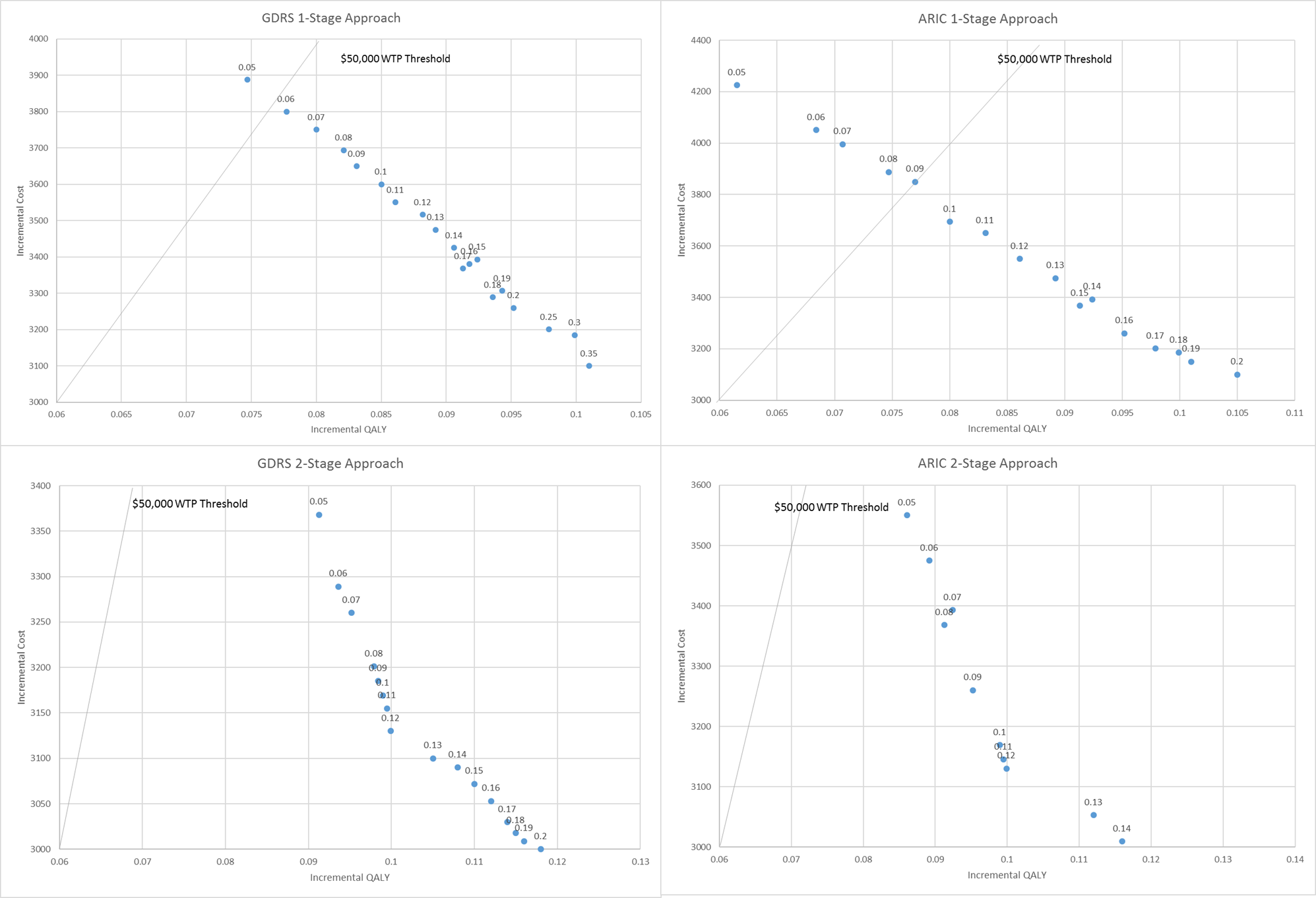
**

**Suppl. Fig. 1 Cost-effectiveness Plane for all scenarios of the base-case analysis.**

**
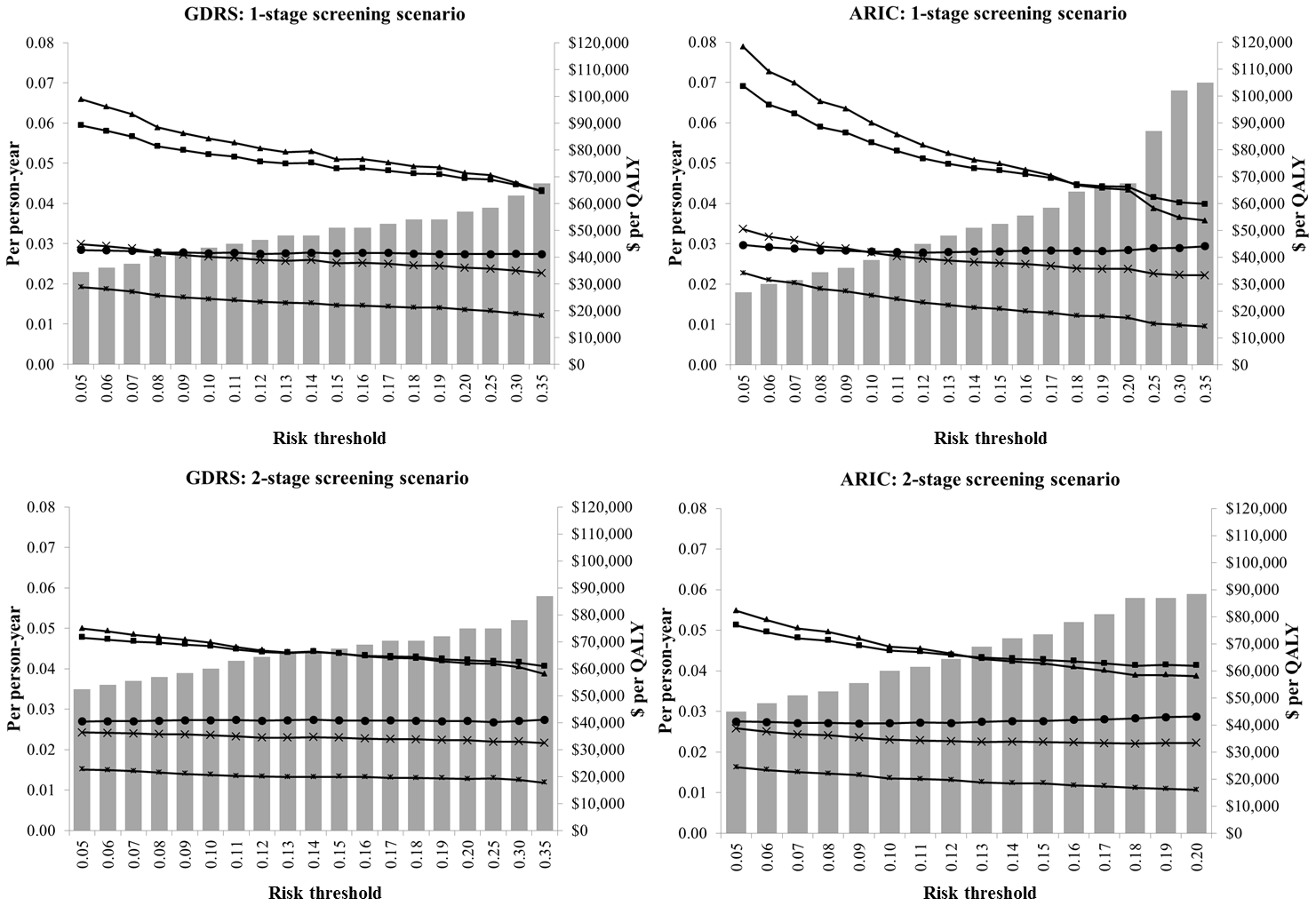
**

**Suppl. Fig. 2 Annual incidence rates and cost per QALY gained (ICER) by risk cut-offs of the GDRS and the ARIC 2009 model in a 1-stage and 2-stage appraoch for five different sensitivity analyses.**

Annual incidence rates were determined in a US population using data from the ARIC and CHS studies. Varying thresholds of diabetes risk were used to stratify the population and diabetes incidence was calculated for the respective high-risk group (≥ risk threshold for 1-stage; ≥ risk threshold and ≥ 100mg/dl glucose for 2-stage). Sensitivity analyses included the following assumptions: double costs (triangle), a lower reduction of relative diabetes risk (by 12.5% and 6.75% after 3 years) due to the intervention (box), a higher reduction of relative diabetes risk (by 50% and 25% after 3 years) due to the intervention (star), stable effectiveness and costs over time (dot) and additional health benefit in hypertension due to the intervention (reduction by 26%) (x).

**
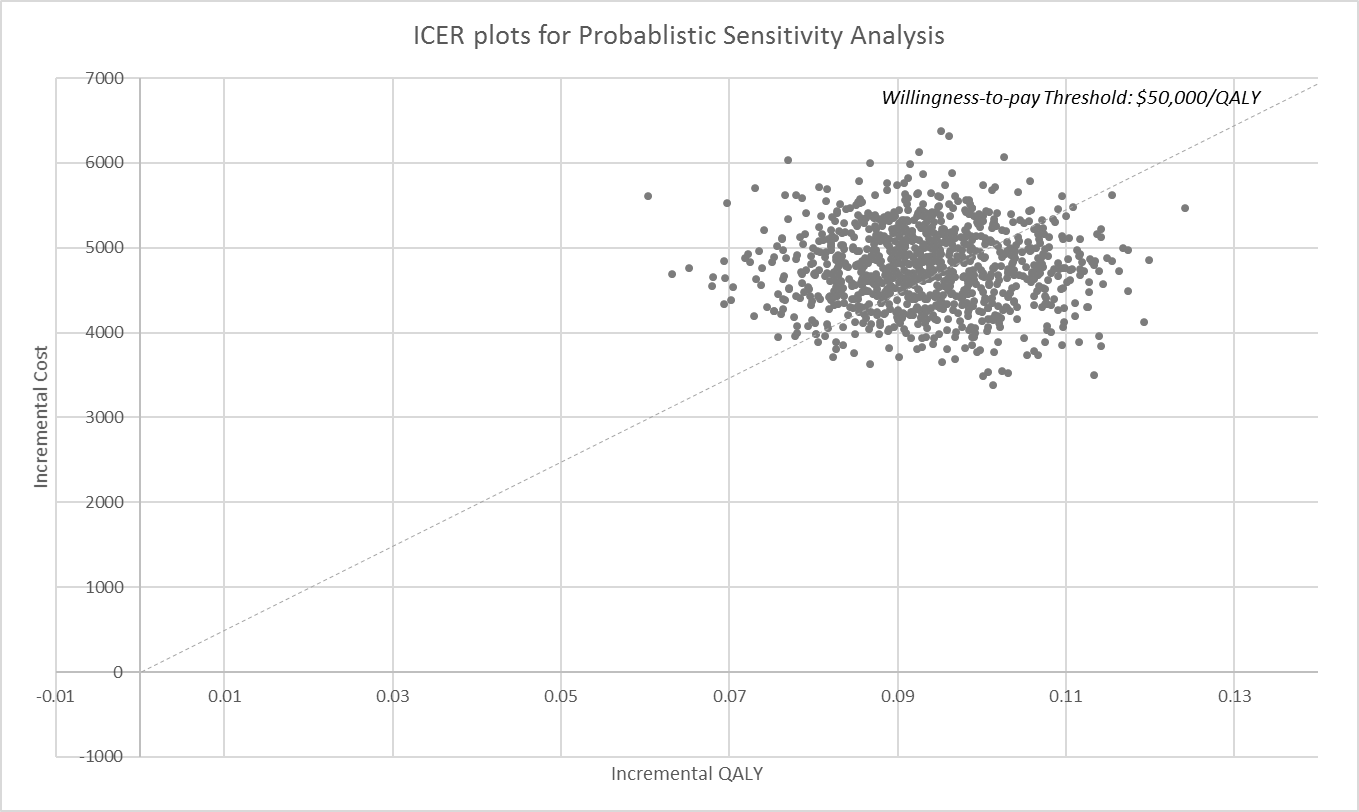
**

**Suppl. Fig. 3. Scatter Plot for Probabilistic Sensitivity Analysis**

**
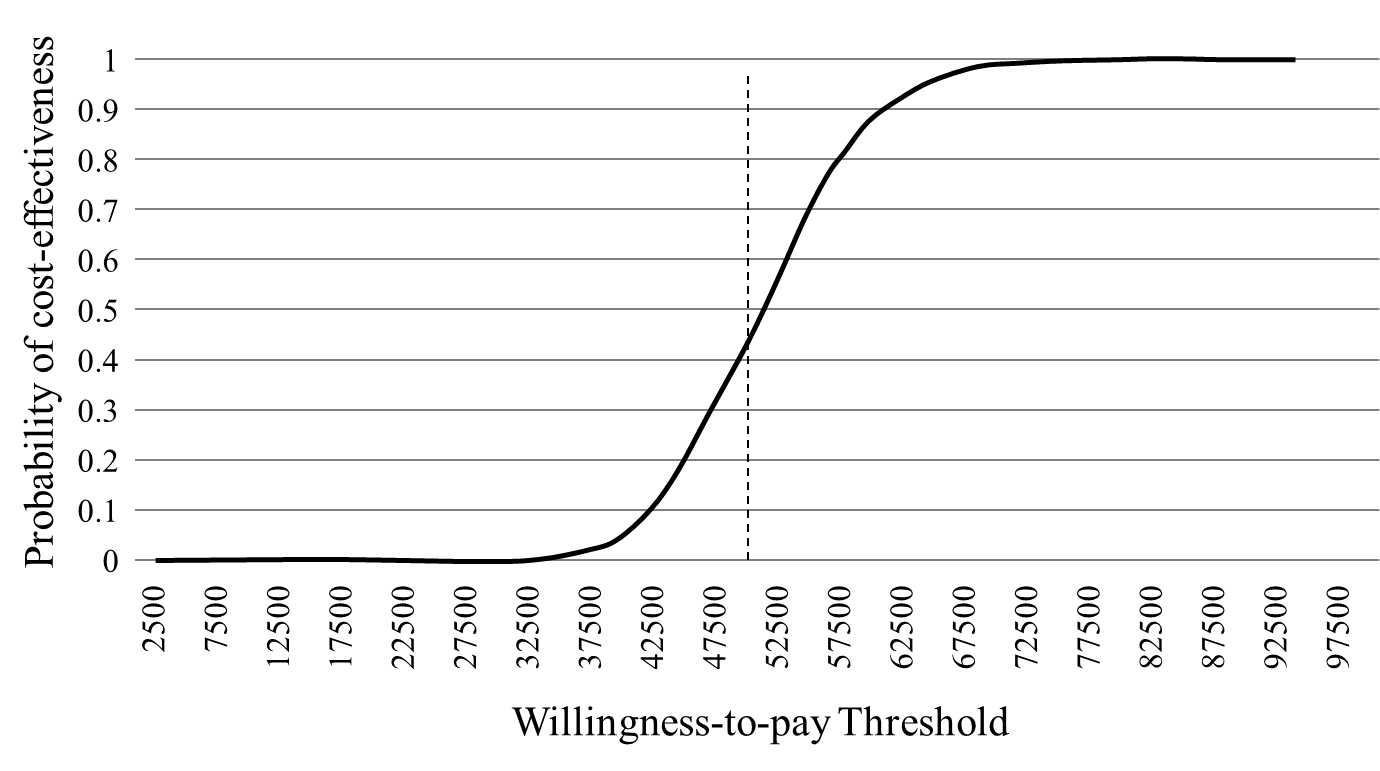
**

**Suppl. Fig. 4. Probabilistic Acceptance Curve**
